# Lung- and Diaphragm-Protective Ventilation by Titrating Inspiratory Support to Diaphragm Effort: A Randomized Clinical Trial

**DOI:** 10.1097/CCM.0000000000005395

**Published:** 2022-02-04

**Authors:** Heder J. de Vries, Annemijn H. Jonkman, Harm J. de Grooth, Jan Willem. Duitman, Armand R. J. Girbes, Coen A. C. Ottenheijm, Marcus J. Schultz, Peter M. van de Ven, Yingrui Zhang, Angelique M. E. de Man, Pieter R. Tuinman, Leo M. A. Heunks

**Affiliations:** 1 Department of Intensive Care Medicine, Amsterdam UMC location VUmc, Amsterdam, the Netherlands.; 2 Amsterdam Cardiovascular Sciences Research Institute, Amsterdam, the Netherlands.; 3 Center for Experimental and Molecular Medicine, Amsterdam UMC, location AMC, Amsterdam, the Netherlands.; 4 Department of Physiology, Amsterdam UMC location VUmc, Amsterdam, the Netherlands.; 5 Department of Intensive Care Medicine, Amsterdam UMC location AMC, Amsterdam, the Netherlands.; 6 Nuffield Department of Medicine, Mahidol University, Bangkok, Thailand.; 7 Mahidol-Oxford Tropical Medicine Research Unit (MORU), Mahidol University, Bangkok, Thailand.; 8 Department of Epidemiology and Data Science, Amsterdam UMC, Location VUmc, Amsterdam, the Netherlands.; 9 Department of Critical Care Medicine, Fujian Provincial Hospital, Fujian Provincial Center for Critical Care Medicine, Fujian Medical University, Fuzhou, Fujian, China.

**Keywords:** critical illness, diaphragm, esophageal pressure measurement, mechanical ventilation, work of breathing

## Abstract

Lung- and diaphragm-protective ventilation is a novel concept that aims to limit the detrimental effects of mechanical ventilation on the diaphragm while remaining within limits of lung-protective ventilation. The premise is that low breathing effort under mechanical ventilation causes diaphragm atrophy, whereas excessive breathing effort induces diaphragm and lung injury. In a proof-of-concept study, we aimed to assess whether titration of inspiratory support based on diaphragm effort increases the time that patients have effort in a predefined “diaphragm-protective” range, without compromising lung-protective ventilation.

**DESIGN:**

Randomized clinical trial.

**SETTING:**

Mixed medical-surgical ICU in a tertiary academic hospital in the Netherlands.

**PATIENTS:**

Patients (*n* = 40) with respiratory failure ventilated in a partially-supported mode.

**INTERVENTIONS:**

In the intervention group, inspiratory support was titrated hourly to obtain transdiaphragmatic pressure swings in the predefined “diaphragm-protective” range (3–12 cm H_2_O). The control group received standard-of-care.

**MEASUREMENTS AND MAIN RESULTS:**

Transdiaphragmatic pressure, transpulmonary pressure, and tidal volume were monitored continuously for 24 hours in both groups. In the intervention group, more breaths were within “diaphragm-protective” range compared with the control group (median 81%; interquartile range [64–86%] vs 35% [16–60%], respectively; *p* < 0.001). Dynamic transpulmonary pressures (20.5 ± 7.1 vs 18.5 ± 7.0 cm H_2_O; *p* = 0.321) and tidal volumes (7.56 ± 1.47 vs 7.54 ± 1.22 mL/kg; *p* = 0.961) were not different in the intervention and control group, respectively.

**CONCLUSIONS:**

Titration of inspiratory support based on patient breathing effort greatly increased the time that patients had diaphragm effort in the predefined “diaphragm-protective” range without compromising tidal volumes and transpulmonary pressures. This study provides a strong rationale for further studies powered on patient-centered outcomes.

## Abstract 2

Supplemental Digital Content is available in the text.

New approaches are needed to limit the adverse effects of invasive mechanical ventilation on the diaphragm of critically ill patients, as diaphragm weakness in these patients is common and has been associated with poor clinical outcomes (1, 2). The level of diaphragm effort has been proposed to play a role in the development of critical illness-associated diaphragm weakness (3): inactivity of the diaphragm causes disuse atrophy and diaphragm weakness (4–6), whereas excessive diaphragm effort has been implicated to contribute to diaphragm injury in observational (5) and preclinical studies (7–9). Prospective clinical trials are required to confirm this hypothesis (10). Additionally, excessive diaphragm effort might worsen lung injury by increasing stress and strain imposed on the lung (self-inflicted lung injury) (11, 12) and by the hemodynamic consequences of large intrathoracic pressure swings (13–15). Preventing low and excessive diaphragm effort by titrating inspiratory support might thus limit the complications associated with mechanical ventilation on the diaphragm and lungs (5, 12, 16).

Lung- and diaphragm-protective mechanical ventilation is a novel concept to managing patients on mechanical ventilation, aimed at achieving physiologic diaphragm effort while remaining within limits of lung-protective ventilation (17, 18). Although incorporating diaphragm effort into management of ventilated patients has gained attention in the past years, the feasibility of this concept and its compatibility with lung-protective ventilation strategies have not been investigated.

We performed a randomized clinical trial to establish the feasibility of a lung- and diaphragm-protective ventilation approach in invasively ventilated, critically ill patients. We hypothesized that titrating inspiratory support to diaphragm effort would increase the time that patients have effort in a predefined “diaphragm-protective” range, without compromising lung-protective ventilation.

## MATERIALS AND METHODS

### Study Design

We performed a randomized clinical trial in a mixed medical-surgical ICU of an academic hospital in the Netherlands. The trial was registered at ClinicalTrails.gov (NCT03527797). The study protocol was approved by the institutional review board (NL62486.029.17). The patient or their legal representatives provided written informed consent. The study was performed in accordance with the 2008 Declaration of Helsinki and its later amendments. No commercial support was received for this project.

### PATIENTS

Adult patients were eligible if they were intubated and mechanically ventilated in a partially supported mode and if the attending physician expected invasive ventilation would be required for at least 24–48 hours at the time of screening. Exclusion criteria were as follows: past medical history of neuromuscular disorders (including diaphragm paralysis), contraindications for placement of a nasogastric catheter, active air leak in the pleural space, or abnormal anatomy of the esophagus or stomach.

### Randomization and Masking

Enrollment, randomization, and clinical data collection were handled in an online system (Castor EDC; Castor, Amsterdam, the Netherlands). Patients were allocated to the control or intervention group in a 1:1 ratio using variable block randomization with blocks of size 4, 6, or 8. Patients and their families were blinded to group allocation. Blinding was not possible for the investigators and the clinical team given the study design. Patients excluded before randomization were replaced.

### Procedures

**Figure 1.**
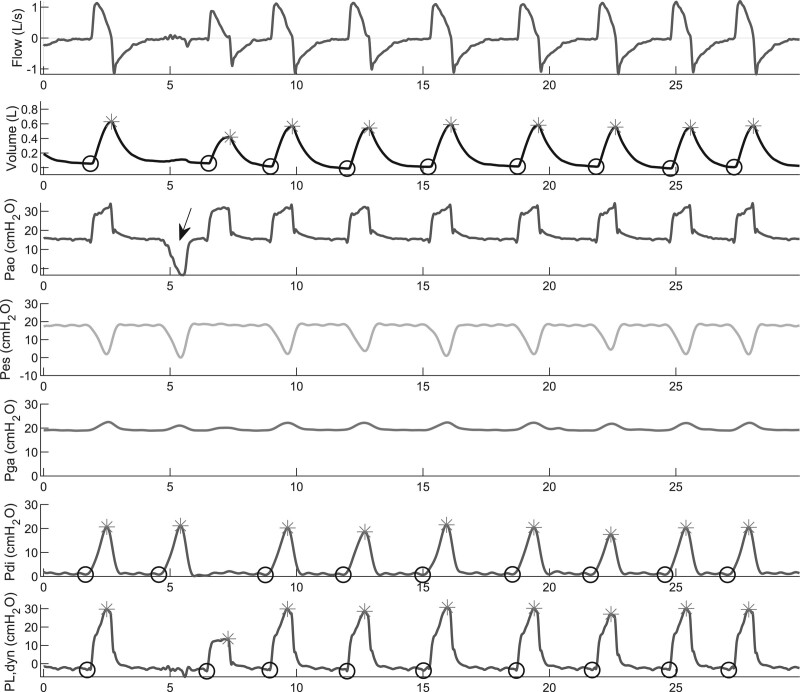
Analysis of the physiologic signals. Flow, volume, airway opening pressure (Pao), esophageal pressure (Pes), gastric pressure (Pga), transdiaphragmatic pressure (Pdi), and transpulmonary pressure (PL,dyn) during the first 30 s of an hour of recordings. An end-expiratory occlusion was administered at the arrow to confirm adequate positioning and filling of the catheter. The *asterisks* mark the maximal volume, Pdi, and P_L_ identified by the script in each breath, respectively, whereas the *circles* mark the minimal values. The delta in each breath was calculated as maximum–minimum (dynamic pressures).

Flow, airway opening pressure (Pao), esophageal pressure (Pes), gastric pressure (Pga), transdiaphragmatic pressure (Pdi, calculated as Pga–Pes), and dynamic transpulmonary pressure (PLdyn, calculated as Pao–Pes) (13) were recorded continuously during the 24-hour study period and stored for later analyses (**Fig. 1**).

The first hour of measurements in both groups (T = 0 hr) was conducted before adjusting the inspiratory support to serve as baseline.

**Figure 2.**
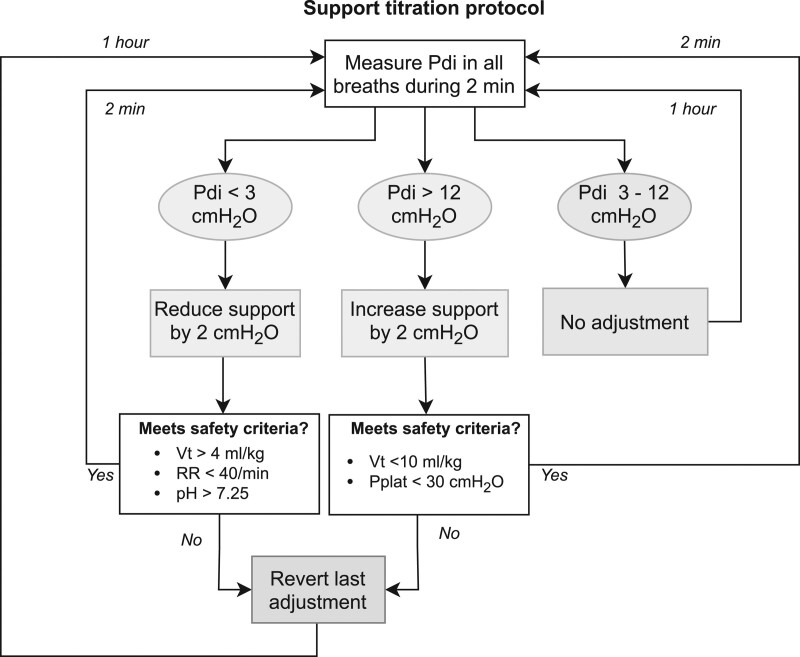
Titration algorithm. An increase in tidal volume greater than 2 mL/kg predicted bodyweight compared with a subject’s own baseline was also considered a breach of lung-protective ventilation. Pdi = transdiaphragmatic pressure, Pplat = plateau airway pressure, RR = respiratory rate, Vt = tidal volume.

Patients in the control group received standard clinical care following local protocols for lung-protective ventilation and sedation (**online supplement**, http://links.lww.com/CCM/G924) from T = 0 hour to T = 24 hours. In the intervention group, ventilatory support was adjusted from T = 1 hour to T = 24 hours based on diaphragm effort according to the algorithm presented in **Figure 2** (“diaphragm-protective ventilation”). A study investigator (H.J.d.V., A.H.J., L.M.H.) measured the mean Pdi in the first 2 minutes of every hour in real time using the data capture software (Acknowledge; BIOPAC, Goleta, CA). The steps of the algorithm were repeated until the mean Pdi was between 3 and 12 cm H_2_O or if a predefined limit for lung-protective ventilation was crossed. The lower limit (3 cm H_2_O) for diaphragm effort was selected because we could reliably differentiate pressure swings of 3 cm H_2_O from cardiac oscillations, and very low diaphragm effort was found to prevent disuse atrophy in animal models (19) and preliminary clinical studies (20, 21). The upper limit (12 cm H_2_O) was based on the upper range of tidal swings in Pes in healthy subjects (13, 22). This range is in agreement with the opinion of a group of international experts published recently (17, 18). The investigators only adjusted the inspiratory support; other ventilator settings (including positive end-expiratory pressure [PEEP], Fio_2_, cycle criteria, trigger settings) and all other aspects of care (including drugs) were managed by the clinical team according to local protocols. Study data were not available to the clinical team. Blood samples (5–10 mL) were drawn from the indwelling arterial catheter at T = 0 hour, T = 12 hours, and T = 24 hours.

### Outcomes

The primary outcome was the proportion of breaths in the “diaphragm-protective” range per patient, calculated as (number of breaths with Pdi swings between 3 and 12 cm H_2_O)/(all recorded breaths) × 100%. Secondary outcome variables included the tidal volume normalized to predicted bodyweight (mL/kg), and the dynamic and driving transpulmonary pressures measured in every breath in the 24-hour study period (Fig. 1). Additional measures of lung-protective ventilation, including the pressure-time product of the diaphragm and the concentrations of protein biomarkers for endothelial function, lung injury, and systemic inflammation are described in the online supplement (http://links.lww.com/CCM/G924).

### Statistical Analysis

A convenience sample of 40 patients (20 per group) was recruited, because the distribution of respiratory effort during a 24-hour period was not well characterized in the target population and because no previous study had titrated diaphragm effort to this specific range. All statistical analyses were performed on the intention-to-treat population, consisting of all randomized patients that had completed at least 1 hour of measurements. Baseline characteristics were summarized as mean ± sd, median (interquartile range [IQR]), or frequency (percentages) as appropriate. Aggregated outcome data were compared between groups using the Wilcoxon rank-sum test or Student *t* test, as appropriate. For the nonparametric variables, the effect size is reported as the difference in medians with bootstrapped 95% CIs. Normality was assessed with normal-probability plots. When required, a suitable transformation was used to achieve normality. A two-tailed significance level of 5% was used for all statistical analysis. All the statistical analyses were performed in R Version 4.0.1 (R Foundation for Statistical Programming, Vienna, Austria). Additional details on the statistical analyses are available in the online resources.

## RESULTS

In total, 451 patients on partially supported ventilation were assessed between April 25, 2018, and July 16, 2020 (**Fig. E1**, http://links.lww.com/CCM/G924). The trial was stopped because the intended number of participants was included. The intention-to-treat analysis included 39 patients (19 intervention, 20 control). Patient characteristics are summarized in **Table 1** and **Table E1** (http://links.lww.com/CCM/G924). The two groups were similar at baseline. Expected hospital mortality based on the Acute Physiology and Chronic Health Evaluation IV score was 45%. Thirty-five patients (90%) met criteria for acute respiratory distress syndrome (ARDS) according to the Berlin definition (23).

**Table 1.** Baseline Characteristics

Variables	Overall (*N* = 39)	Control (*N* = 20)	Intervention (*N* = 19)
Biometrics			
Age, yr, mean (sd)	65 (14)	66 (14)	65 (13)
Gender = male, *n* (%)	26 (68)	13 (65)	13 (68)
Body mass index kg/m^2^, median (IQR)	27 (26–29)	28 (26–30)	26 (25–28)
Risk scores			
Simplified Acute Physiology Score II, mean (sd)	50 (12)	51 (13)	49 (11)
Sequential Organ Failure Assessment score at enrollment, median (IQR)	9 (8–11)	9 (8–10)	10 (9–12)
Acute Physiology and Chronic Health Evaluation IV, mean (sd)	85 (28)	84 (30)	87 (26)
Mechanical ventilation			
Ventilation prior to study, d, median (IQR)	8 (4–15)	8 (4–15)	9 (5–16)
Controlled ventilation, median (IQR)	3 (1–5)	3 (1–4)	3 (1–8)
Partially supported ventilation, median (IQR)	4 (2–10)	4 (2–10)	3 (2–9)
PEEP, cm H_2_O, median (IQR)	10 (8–12)	10 (8–12)	10 (8–10)
Pressure above PEEP, cm H_2_O, mean (sd)	9.5 (4.8)	8.5 (4.7)	10.7 (4.8)
Fio_2_, median (IQR)	0.45 (0.40–0.50)	0.45 (0.40–0.50)	0.45 (0.40–0.50)
Gas exchange, mean (sd)			
pH	7.42 (0.08)	7.42 (0.08)	7.42 (0.07)
Pao_2_, mm Hg	79.5 (13.5)	78.8 (15.0)	79.5 (12.8)
Paco_2_, mm Hg	45.0 (9.0)	44.2 (8.2)	45.0 (10.5)
Pao_2_/Fio_2_ ratio, mm Hg	190 (54)	185 (50)	198 (60)
Ventilatory ratio	2.1 (0.6)	2.1 (0.6)	2.1 (0.6)
Respiratory mechanics			
Compliance of respiratory system, mL/cm H_2_O, median (IQR)	36 (23–40)	33 (23–41)	35 (26–47)
Lung compliance, mL/cm H_2_O, median (IQR)	48 (28)	45 (29–64)	48 (33–71)
Chest wall compliance, mL/cm H_2_O, mean (sd)	150 (57)	143 (58)	159 (56)
Intrinsic PEEP, cm H_2_O, mean (sd)	2.6 (2.2)	2.3 (1.7)	2.9 (2.7)
Neurologic, median (IQR)			
Richmond Agitation and Sedation Score at enrollment	–1 (–3 to 0)	–1 (–2 to 0)	–2 (–3 to 0)

IQR = interquartile range, PEEP = positive end-expiratory pressure.

### Inspiratory Support Adjustments

Inspiratory support was adjusted a median of 2 (IQR, 1–2) times per subject in the control group (by the clinical team) and median of 8 (IQR, 4–11) times per subject in the intervention group (by the investigators according to the titration algorithm) in the 24-hour study period (*p* < 0.001) (**Fig. E2**, http://links.lww.com/CCM/G924). Most of the adjustments in the intervention group (52%) were required in the first 4 hours of the study period (**Fig. E3**, http://links.lww.com/CCM/G924), and most subjects received a net increase in support (median 3 cm H_2_O; IQR, 2–6 cm H_2_O). Median difference in PLdyn was equal in both groups, with two notable outliers in the intervention group (Fig. E2, http://links.lww.com/CCM/G924).

### Diaphragm Effort

More than 1 million breaths were analyzed for the primary and secondary outcome variables (mean 28,894 ± 9,796 breaths per subject). Seventy-two hours (7.7% of the total) had missing data (online supplement, http://links.lww.com/CCM/G924). At baseline, 7% of breaths had effort below and 37% of breaths had effort above the target range (**Fig. E4**, http://links.lww.com/CCM/G924).

**Figure 3.**
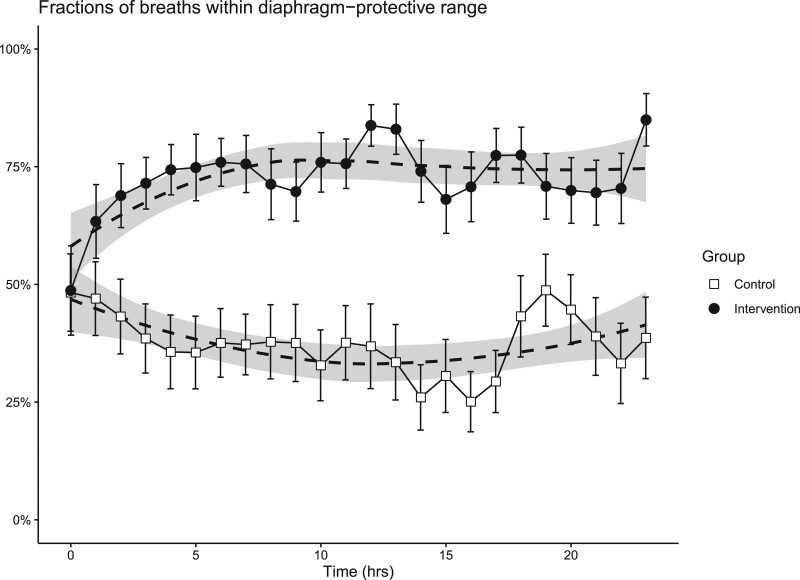
Proportion of breaths in diaphragm-protective range, defined as 3–12 cm H_2_O per breath, in each group. *Dots* represent the mean; *bars* represent the se of the mean; *asterisks* represent the hours with a significant difference between the groups in the post hoc analysis. Shaded area represents the 95% CI obtained with Loess regression.

The evolution of diaphragm effort from T = 0 hour to T = 24 hours is shown in **Figure 3**. Proportions of breaths within the target range of diaphragm effort, summarized over the total study period, were higher for patients in the intervention group compared with patients in the control group (median 81% [64–86%] vs 35% [16–59%], respectively, difference in median 46%; 95% CI, 24–64%; *p* < 0.001). The longitudinal course differed significantly between the groups (*p* < 0.001). Post hoc subgroup analyses showed that the inspiratory support titration was equally effective in patients with a compliance below and above the median (35 mL/cm H_2_O) and in patients included within 7 days after onset of ventilation or later (**Table E2**, http://links.lww.com/CCM/G924). Distribution of breaths below and above the target range for diaphragm effort, pressure-time product of the diaphragm, and patient-level data on diaphragm effort are available in the online supplement (**Figs. E5–E7**, http://links.lww.com/CCM/G924**).**

### Markers for Lung Injury

**Figure 4.**
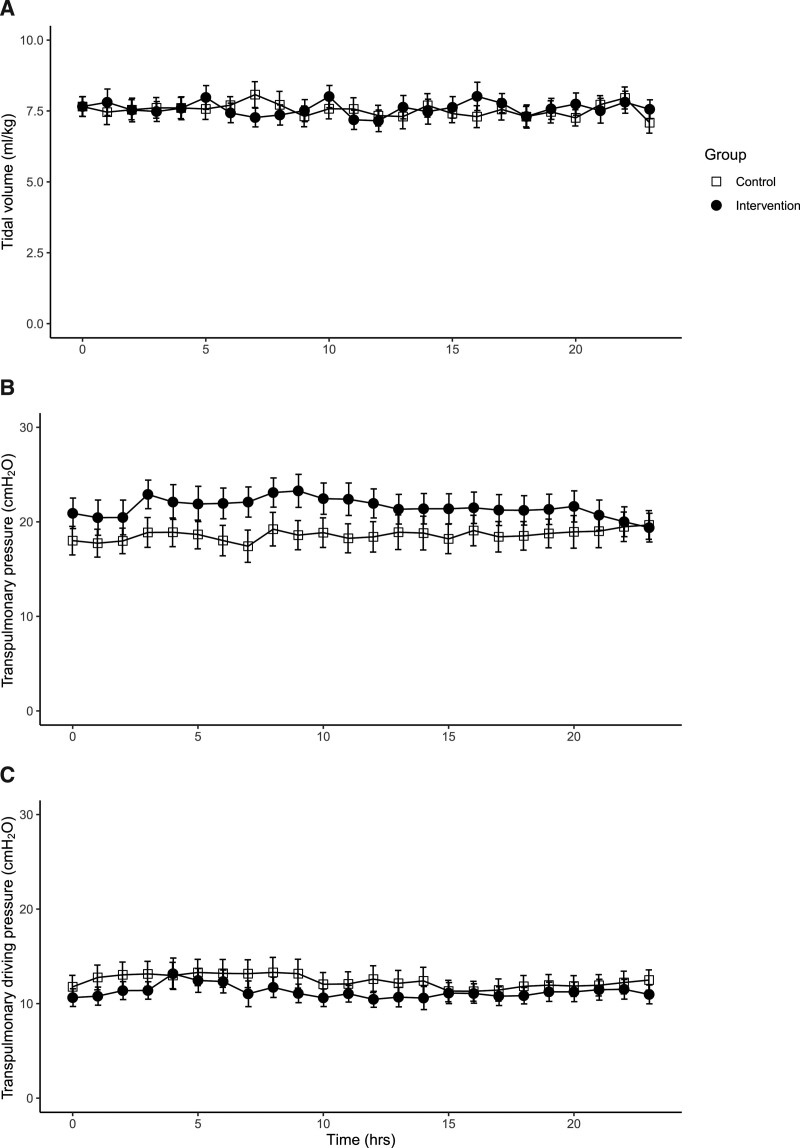
Tidal volume (**A**), dynamic transpulmonary pressures (**B**)‚ and transpulmonary driving pressures (**C**) over time. *Dots* represent the mean; *bars* represent the sem. None of the hours differed significantly between both groups in the post hoc analysis.

Tidal volumes were similar in the intervention and control groups (7.56 ± 1.47 vs 7.54 ± 1.22 mL/kg predicted body weight; *p* = 0.959) (**Fig. 4*A***). The proportion of breaths in lung-protective range, defined as (number of breaths with tidal volumes < 8 mL/kg)/(all breaths), was found to be similar in the intervention and control groups (median 96% [54–99%] vs 83% [35–86%], respectively; *p* = 0.255) (**Fig. E8**, http://links.lww.com/CCM/G924) in a post hoc analysis. The cutoff (< 8 mL/kg) was based on a recent expert statement (17).

The PLdyns, the sum of pressures used to overcome airflow resistance and elastance of the lungs, were similar in the intervention and control groups (20.5 ± 7.1 vs 18.5 ± 7.0 H_2_O cm H_2_O, respectively; *p* = 0.373) (**Fig. 4*B***). The transpulmonary “driving” pressure, the pressure used to overcome the elastance of the lungs, was measured in 28 subjects and calculated in 11 subjects in a post hoc analysis (online supplement, http://links.lww.com/CCM/G924). The transpulmonary driving pressures were similar in the intervention and control groups (11.2 ± 5.6 vs 12.4 ± 4.4 cm H_2_O, respectively; *p* = 0.295) (**Fig. 4*C***).

The doses of sedatives, pH, Paco_2_, and Pao_2_ did not differ between the groups (**Table E3**, http://links.lww.com/CCM/G924). Longitudinal course of 12 protein biomarkers of lung endothelial cell function, lung injury and systemic inflammation, did not differ between the groups for any of the tested biomarkers (**Table E4**, http://links.lww.com/CCM/G924). Longitudinal course of minute volume was not different in both groups (**Fig. E9**, http://links.lww.com/CCM/G924).

### Patient Outcomes and Adverse Events

Weaning outcome and mortality were similar in both groups (**Table E5**, http://links.lww.com/CCM/G924). One subject developed subcutaneous emphysema 10 hours after study titration commenced. Subcutaneous emphysema did not lead to cardiovascular or ventilatory complications and resolved without a chest tube. Severity of the event was categorized as mild. More information is available in the online supplement (**Fig. E10**, http://links.lww.com/CCM/G924).

## DISCUSSION

This is the first study to investigate the feasibility and efficacy of a bedside titration algorithm to obtain diaphragm effort in a predefined “diaphragm-protective” range in a heterogeneous group of invasively ventilated critically ill patients, while maintaining tidal volume and transpulmonary pressures in ranges considered as lung protective. We found that titration of ventilatory support guided by Pdi resulted in higher proportions of breaths in the predefined “diaphragm-protective range” compared with standard of care (81% vs 35%, respectively). This approach did not compromise key characteristics of lung-protective ventilation, including tidal volumes, transpulmonary pressures, and biomarkers for lung injury.

### Diaphragm Effort, Diaphragm Weakness, and ICU Outcomes

The evidence for disuse atrophy caused by ventilator over-assist in critically ill patients is convincing (24, 25). However, load-induced diaphragm injury is an attractive concept but is not yet supported by strong evidence. Animal studies have demonstrated that loaded breathing during mechanical ventilation can induce diaphragm injury, but the load imposed was generally very high (8, 26, 27). Also, it has been demonstrated that high inspiratory loading is associated with diaphragm sarcomeric disruption (7), indicating that the diaphragm is susceptible to load-induced injury. Interestingly, we have reported sarcomeric injury in the diaphragm of ventilated ICU patients (6, 28). Finally, high diaphragm contractile activity assessed indirectly with ultrasound was associated with increases in diaphragm thickness in an observational study (1, 5). Whether the increased diaphragm thickness is reflects muscle injury remains to be investigated. For more extensive discussion, we refer to recent articles (3, 10). Second, the relationship between diaphragm weakness and ICU outcomes, including difficult weaning and ICU mortality, has been observed to various degrees in observational studies (2, 29–35), but whether diaphragm weakness is a causal contributor to poor ICU outcomes or a merely a marker for disease severity remains to be established (36). A causal relationship seems plausible, as the diaphragm is the main muscle of inspiration and improving diaphragm strength led to improved weaning outcome in selected patients (37). A recent mediation analysis of observational data has strengthened the hypothesis that inappropriate diaphragm effort contributes to poor clinical outcomes (10). Nevertheless, large interventional trials that target optimization of diaphragm effort are required to assess the whether inappropriate diaphragm effort leads to diaphragm “myotrauma” and poor outcomes, and this current study might aid in designing such trials.

### Effectiveness of the Titration Algorithm

At baseline, 49% of the subjects (19/39) had insufficient or excessive diaphragm effort according to our predefined limits (**Fig. E3**, http://links.lww.com/CCM/G924). The high occurrence rate of excessive diaphragm effort matches an observational study in patients on partially supported mechanical ventilation (38). Other cohorts have found more patients with low respiratory effort (5). The lower proportion of patients with ventilator over-assistance in our study may be explained by our local clinical protocol that promotes reducing inspiratory support as much as tolerated by the patient. The titration algorithm effectively prevented both insufficient and excessive diaphragm effort in the intervention group; 10% of the subjects (2/19) in the intervention group had diaphragm effort outside the predefined range in the total study period versus 60% (12/20) in the control group (**Fig. E5**, http://links.lww.com/CCM/G924). Notably, this reduction in excessive diaphragm effort was achieved without changing the level of sedation.

### Markers for Lung Injury

The tidal volumes in both groups of our trial closely match the tidal volumes reported for patients on partially-supported mechanical ventilation with ARDS (39) and without ARDS (40) in large observational cohorts (40), demonstrating that the titration algorithm did not compromise lung-protective ventilation. This is further supported by the observation that biomarkers for lung injury and systemic inflammation did not differ significantly between the control and intervention groups during the course of the study. Application of the titration algorithm did not lead to lower diaphragm effort at all in two subjects in the intervention group and instead led to tidal volumes incompatible with lung-protective ventilation. Interestingly, both subjects had a pH greater than 7.48, suggesting that their respiratory drive did not originate from pH and Paco_2_. Their elevated drive might have originated from mechano- and irritant-receptors in the alveoli and chest wall, or pain and agitation. Instead of increasing support, these patients might require sedatives, analgesics, or partial neuromuscular blockade to achieve lung- and diaphragm-protective ventilation (41).

### Strengths

This is the first randomized clinical trial to investigate the feasibility of a lung- and diaphragm-protective ventilation approach in ventilated critically ill patients. The target range for diaphragm effort that we selected is in agreement with the opinion of a group of international experts published recently (17, 18). Additionally, we used the reference standard to measure diaphragm effort (42) and employed a detailed analysis of every single breath in the 24-hour study period. Although the study was single blinded, the clinical team did not have access to results from Pes monitoring. We used a simple algorithm to titrate inspiratory ventilatory support to achieve respiratory effort within physiologic limits without modifying sedation levels, because higher sedation levels are associated with delirium and prolonged mechanical ventilation (43).

### Limitations

This study has several limitations. First, this study was not designed to detect a meaningful impact of “diaphragm-protective” ventilation on diaphragm function, markers for lung injury, or patient outcomes but instead focused on the feasibility of such an approach and its compatibility with lung-protective ventilation. The relatively small number of subjects allowed us to collect in-depth physiologic data but restricted the analysis to physiologic variables. Future studies will have to assess whether this approach indeed reduces the development of diaphragm weakness and improves ICU outcomes. Second, the precise range of diaphragm effort to prevent both disuse atrophy and load-induced injury remains to be established. Especially the upper limit for safe diaphragm effort is subject of discussion and probably depends on several factors including patient characteristics, such as maximal diaphragm strength (44) and the phase of critical illness (33). Third, the study population was heterogeneous. Patients varied considerably in the duration of mechanical ventilation before study inclusion and in their respiratory system compliance. Nevertheless, additional analyses revealed that the titration algorithm was equally effective in patients with a compliance below and above 35 cm H_2_O and in patients included in the first week of ventilation or thereafter (Table E2, http://links.lww.com/CCM/G924). Future studies may start inspiratory support titration as soon as a patient exhibits respiratory effort, as in the early phase of critical illness, the diaphragm may be more susceptible to injury (3, 5). Additionally, the total duration of ventilation and the reintubation rate were high in our cohort because we selected patients in whom prolonged mechanical ventilation was expected. However, it can be argued that this is the population in which protection of the diaphragm can have most impact. Fourth, the study protocol requires Pes and Pga monitoring, which limits generalizability. Recent reports evaluated readily available metrics of respiratory effort based on airway pressure, including the P0.1 and the airway occlusion pressure during a full breath (38, 45). If further research has validated these indirect measurements of respiratory muscle effort, and when the optimal range of effort is better defined, they may be useful to screen for patients who can benefit from invasive measurement techniques (46). Fifth, additional ventilator settings such as the cycle-off criterion, trigger sensitivity, Fio_2_, and PEEP could have been incorporated in the algorithm as these variables influence diaphragm effort (10). However, the role of these settings in lung injury and diaphragm dysfunction is currently less established (3, 10).

## CONCLUSIONS

We found that titration of inspiratory support guided by Pdi increases the time that patients have diaphragm effort in a predefined “diaphragm-protective” range without compromising lung-protective ventilation. Larger trials are required to establish the clinical impact of titrating diaphragm effort on patient-centered outcomes.

## ACKNOWLEDGMENTS

We would like to thank all patients for their valuable contributions, R. H. Driessen for data management support, T. Dekker and B. Dierdorp from the Department of Experimental Immunology for their technical support in the execution and analysis of the biomarker assay, and T. van de Poll for advice on the protein biomarker analysis.

## Supplementary Material


