# Supplementary material for: Lung- and Diaphragm-Protective Ventilation by Titrating Inspiratory Support to Diaphragm Effort: A Randomized Clinical Trial
Source: Crit Care Med. 2022 Feb 4;50(2):192–203. doi: 10.1097/CCM.0000000000005395 (PMC8797006; doi:10.1097/CCM.0000000000005395)
Supplement: Supplementary file 1 [file ccm-50-0192-s001.docx]

**Lung- and diaphragm-protective ventilation by titrating inspiratory support to diaphragm effort: a randomized clinical trial**

*Supplemental Digital Content 1: supplementary methods and results*

Critical Care Medicine, 2021

Heder de Vries,^1,2^ Annemijn Jonkman^1,2^, Harm Jan de Grooth^1^, Jan Willem Duitman^3^, Armand Girbes^1^, Coen Ottenheijm^2,4^, Marcus Schultz^5-7^, Peter van de Ven^8^_,_ Yingrui Zhang^1,9^, Angelique de Man^1^, Pieter Roel Tuinman^1^, Leo Heunks^1,2^

*Affiliations:*

1: Department of Intensive Care Medicine, Amsterdam UMC location VUmc, Amsterdam, The Netherlands

2: Amsterdam Cardiovascular Sciences Research Institute, Amsterdam, the Netherlands
3: Center for Experimental and Molecular Medicine, Amsterdam UMC, location AMC, Amsterdam, the Netherlands

4: Department of Physiology, Amsterdam UMC location VUmc, Amsterdam, the Netherlands
5: Department of Intensive Care Medicine, Amsterdam UMC location AMC, Amsterdam, the Netherlands

6: Nuffield Department of Medicine, Mahidol University, Bangkok, Thailand

7: Mahidol-Oxford Tropical Medicine Research Unit (MORU), Mahidol University, Bangkok, Thailand
8: Department of Epidemiology and Data Science, Amsterdam UMC, Location VUmc, Amsterdam, the Netherlands

9: Department of Critical Care Medicine, Fujian Provincial Hospital, Fujian Provincial Center for Critical Care Medicine, Fujian Medical University, Fuzhou, Fujian, China

*Correspondence to*: Prof Leo Heunks, MD, PhD. Amsterdam UMC, location VUmc, Postbox 7505, 1007 MB Amsterdam, The Netherlands. E-mail: L.Heunks@amsterdamumc.nl

# **Supplementary methods**

**Standard care**Standard care followed local clinical guidelines for ventilation, sedatives and infection prevention, and was performed by independent board-certified ICU physicians and ICU nurses not involved in the trial. The nurse-to-patient ratio was 1:1 tot 1:2, depending on severity of illness. Physicians and nurses applied restrictive sedation targeting analgo-sedation over hypno-sedation. ICU nurses performed standard airway care, inclusion endotracheal suctioning, when required.

Inspiratory support was set to tidal volumes of 6 – 8 ml/kg predicted bodyweight, and respiratory rates between 20 and 30 breaths per minute. Ventilators did not use tube compensation. Lower tidal volumes were prioritized over normocapnia. Trigger sensitivity was set to the lowest value that did not lead to auto-triggering. Cycle criteria was set to 30% of peak inspiratory flow and adjusted if intrinsic PEEP or double-triggering was observed. PEEP was set to the lowest PEEP required to obtain the highest respiratory system compliance. FiO_2_ was adjusted to obtain peripheral SaO_2_ between 90 and 95%. Pressure support was reduced by 2 cmH_2_O each day to stimulate respiratory muscle training, unless this led to an unacceptable increase in PaCO_2_ or agitation. Recruitment maneuvers were not performed routinely, but could be performed (by increasing PEEP to 30-40 cmH_2_O for 40 seconds) if deemed necessary by the attending physician.

The attending physicians assessed patients daily on whether they were able to undergo a trial of spontaneous breathing. Criteria used to assess patients were: PEEP below 10 cmH_2_O, FiO2 below 0.5, hemodynamic stability (no or low-dose of vasopressors and/or inotropes), adequate airway clearance by the patient, and adequate level of consciousness (patient can follow simple commands). If the attending physician deemed a patient ready for extubation, a trial of spontaneous breathing was performed using 5 PEEP and no support, or a t-piece in patients with suspected left ventricular dysfunction.

**Data collection**Patients were ventilated with a Servo-U ventilator (Getinge, Sweden) and instrumented with a dedicated nasogastric catheter (Nutrivent, Sidam, Italy) to measure esophageal pressure (Pes) and gastric pressure (Pga). A differential flow sensor (Adult single-use flow sensor, Hamilton Medical, Switzerland) was placed in series with the ventilator circuitry to measure flow and airway opening pressure (Pao). The pressure and flow sensors were connected to dedicated amplifiers (DA100C, BIOPAC, USA) and to an analog-to-digital converter (MP160, BIOPAC, USA). A dedicated software (Acqknowledge, BIOPAC, USA) stored the signals at 250 Hz on a portable computer. Transdiaphragmatic pressure (Pdi) was calculated as Pga – Pes, dynamic transpulmonary pressure (P_L_) as Pao – Pes, and volume as the time-integral of flow_._(E1) Pao, flow, Pes, Pga, Pdi and P_L_ signals were available for the investigator at the bedside. The raw recordings were used for the titration algorithm in the intervention group. Data were stored offline for further analysis of study outcomes per breath. Correct positioning and filling of the catheter was confirmed with an end-expiratory occlusion as described.(E2) Correct filling of the pressure balloons was assessed hourly and adjusted if necessary. An end-inspiratory occlusion was performed for 3 seconds at baseline to calculate the respiratory system compliance, lung compliance and chest wall compliance.(E3) From subject 12 and onwards, an end-inspiratory occlusion for 3 seconds was performed hourly. Richmond Agitation and Sedation Scale (RASS)-score and the doses of sedatives were collected hourly. Blood gases were collected when required for clinical purposes.

Patient characteristics were collected by chart review when informed consent was obtained. Clinical outcomes were collected by chart review four weeks after completion of all study measurements, and included ICU mortality, hospital mortality, duration of invasive mechanical ventilation, ventilator-free days at day 28, and reintubation rate, which we assessed to characterize the study population. Ventilator-free days were defined as calendar days without assisted breathing for at least 24-consective hours from the date of the last successful extubation until day 28 of admission; all patients died by day 28 were considered to have no ventilator-free days. Patient-ventilator interaction and plateau airway pressures had not been assessed at the time of writing and will be reported later.

### **Analysis of the continuous physiological data**

Offline data processing and analysis was performed using an in-house developed software algorithm (MathLab, Mathworks, USA). The code for this software will be made available in the online resources. Raw data were pre-processed to remove high frequency (>2 Hz) and low frequency (<0.1 Hz) noise before breath-by-breath analysis of diaphragm effort and respiratory mechanics was performed. The software identified each individual breath based on the flow and airway-pressure signals. The tidal swing in Pao, Pes, Pga, Pdi, P_L_, and volume were obtained by subtracting the minimal value from the maximal value obtained in each window with a peak-finding algorithm as shown in **Figure 1** in the main manuscript. Intrinsic PEEP (PEEPi) was calculated as the drop in esophageal pressure before the onset of positive flow in each breath.(E4) The pressure-time product of the diaphragm (PTPdi) was calculated as the time-integral of transdiaphragmatic pressure in each breath, measured from the beginning of the rise in Pdi until the Pdi was at the baseline again, and was calculated with the unit cmH_2_O*s per minute.
Transpulmonary driving pressures were computed by administering a short end-inspiratory and end-expiratory occlusion at the start of every other hour from subject 12 and onwards (**Figure E1)**. Next, lung compliance (CL) was calculated as: CL = Tidal Volume (Vt) / (transpulmonary plateau pressure – end-expiratory transpulmonary pressure). The tidal volume of each breath was used to calculate the driving pressure of the breath as PLdrive = Vt / CL. This method assumes that lung compliance does not vary within two hours. In the first 11 subjects, we have only administered an end-inspiratory occlusion at baseline, and we used the lung compliance obtained at baseline during all subsequent hours.

### **Protein biomarker measurement**

Blood samples were drawn in a plastic tube filled with EDTA and immediately centrifuged at 1800 RPM for 10 minutes (Universal 320R, Hettich, Germany), after which the plasma was extracted and stored at -80° for later analysis. The samples were thawed and measured in a single batch after completion of all the study measurements. The following biomarkers involved in the main pathophysiological pathways of lung injury(E5) were measured using a custom Human Magnetic Luminex Assay (#LXSAHM, R&D systems, MN, USA) according to the manufacturer instructions: Angiopoietin-1, Angiopoietin-2, E-selectin, Intercellular Adhesion Molecule (ICAM)-1, Interleukin (IL)-6, IL-8, IL-10, Receptor for Advanced Glycation End products (RAGE), Surfactant protein (SP)-D, Tumor Necrosis Factor (TNF)-α, Vascular Cell Adhesion Molecule (VCAM)-1 and von Willebrand Factor (vWF)-a2. Values of samples that reached the upper limit of quantification were set to the upper limit of quantification for the specific assay. Measurements that were out of range in the lower limit were set to half of lower limit of quantification.

**Statistical analyses**

Additional analyses were performed to test at which follow-up moments outcomes differed between the groups. For these analyses, outcomes were aggregated over 1-hour periods. Longitudinal course of outcomes was subsequently compared between the groups using a linear mixed model. The linear mixed model included fixed effects for follow-up time (as a categorical covariate), treatment group and their two-way interaction, and a random effect for subject. The longitudinal course was concluded to differ between the groups when the two-way interaction term was significant. In case of a significant two-way interaction, post-hoc tests with a Bonferroni correction were performed to compare the outcomes between treatment groups at each specific hour of follow-up. For the post-hoc subgroup analyses, the mixed models additionally included a categorical covariate for compliance (dichotomized to either below or above 35 ml/cmH_2_O) or the duration of ventilation before admission (dichotomized to either less than or more than 7 days of ventilation before admission), and their two-way interaction terms with time.

Normality of residuals was assessed visually by means of normal-probability plots. When required, a suitable transformation was used to achieve normality of residuals. Missing outcomes were assumed to be missing at random. The assumption was checked visually using line-plots with outcomes plotted as function of follow-up time for each individual. Under the missing at random assumption, the linear mixed model provides unbiased estimates for the treatment effects. A similar procedure was used for biomarkers and outcomes measured at baseline, 12 hours follow-up and 24 hours follow-up.

# **Supplementary Results**

**Calibration and filling**
Adequate esophageal pressure measurements (Pao/Pes-ratio between 0.8 – 1.2) were obtained in all participants at baseline. The esophageal and gastric pressure balloons were refilled on average 4±2 times per subject in the 24-hour study period.

**Missing data:** 30 subjects completed the full 24-hour measurement period. 72 hours (7.7%) of the data were missing in nine subjects (6 control vs 3 intervention): two subjects (1 control and 1 intervention) because of clinical deteriorations unrelated to the study which required them to be ventilated in a pressure control mode; one patient in the intervention group because of technical problems with the measurement setup; and 6 subjects (5 control and 1 intervention) for procedural reasons (e.g., CT scan or bronchoscopy). 7 of the subjects with missing data had completed at least 15 hours of the measurements, and two patients had less than 12 hours of measurements available. Data were found to be ‘missing at random’. All of the available recordings for each subject were used for the primary analysis.

|  | **Overall (n = 39)** | **Control (n = 20)** | **Intervention (n = 19)** | |
| --- | --- | --- | --- | --- |
| **Respiratory mechanics** |  |  |  |  |
| Tidal volume, ml | 520 (130) | 512 (145) | 527 (116) |  |
| Tidal volume normalized to ideal bodyweight, mL/kgPBW | 7·5 (1·8) | 7·4 (2) | 7·6 (1·7) |  |
| Breaths with tidal volume <8 ml/kg, % | 90 [40-100] | 81 [43- 95] | 95 [23-100] |  |
| Dynamic transpulmonary pressure, cmH_2_O | 18 [13-26] | 18 [14-22] | 23 [14-26] |  |
| **Breathing effort** |  |  |  |  |
| Respiratory rate, #/min | 24·8 (6·6) | 25·2 (6·7) | 24·4 (6·7) |  |
| P0·1, cmH_2_O per breath | 3·3 (1·7) | 2·9 (1·6) | 3·8 (1·9) |  |
| ΔPdi, cmH_2_O per breath | 11·3 (5·7) | 9·8 (6·0) | 11·7 (5·6) |  |
| PTPdi, cmH_2_O*s/min | 165 [113-265] | 150 [109-253] | 190 [127-265] |  |
| **Reason for intubation, n (%)** |  |  |  |  |
| Reduced level of consciousness | 1 (3%) | 0 (0%) | 1 (5%) |  |
| Hypoxic respiratory failure | 38 (97%) | 20 (100%) | 18 (95%) |  |
| Cardiogenic | 3 (8%) | 1 (5%) | 2 (11%) |  |
| ARDS | 35 (90%) | 19 (95%) | 16 (84%) |  |
| Pulmonary | 21 (54%) | 9 (45%) | 12 (63%) |  |
| Non-pulmonary | 14 (36%) | 10 (50%) | 4 (21%) |  |

**Table E1.** **Reasons for intubation, respiratory mechanics and effort prior to study**. Proportions may not total 100% due to rounding. Pdi: transdiaphragmatic pressure. PTPdi; transdiaphragmatic pressure-time product. P0·1, airway occlusion pressure during the first 100 ms.

| **Model** | **Parameter** | **p-value** |
| --- | --- | --- |
| Base model | Intercept | 0.323 |
|  | Time | 0.816 |
|  | Group | <0.001 |
|  | Time : Group | <0.001 |
| Model including compliance at baseline | Intercept | 0.323 |
|  | Time | 0.819 |
|  | Group | <0.001 |
|  | Compliance | 0.796 |
|  | Time : Group | <0.001 |
|  | Time : Compliance | 0.607 |
| Model including ventilator-days at baseline | Intercept | 0.323 |
|  | Time | 0.827 |
|  | Group | <0.001 |
|  | Ventilation days | 0.299 |
|  | Time : Group | <0.001 |
|  | Time : Ventilation days | 0.976 |

**Table E2:** Subgroup analysis with mixed linear models. The fraction of breaths within range was transformed with a logit-link to obtain a normal distribution. Compliance was dichotomized according to the median compliance in the population, 35 ml/cmH_2_O, and included as a factor. Days of mechanical ventilation before inclusion was dichotomized to inclusion within the first week of ventilation or after the first week of ventilation, and included as a factor in the model. Additionally, the two-way interaction terms of Group and Time, Compliance and Time and Ventilation days and Time were included to assess their influence on the effectiveness of the titration protoco

| **Parameter** | **Group** | **Time = 0hrs** | **Time = 12hrs** | **Time = 24hrs** | **p*** |
| --- | --- | --- | --- | --- | --- |
| RASS | Intervention | -2 [-3, 0] | -1 [-3, 0] | -2 [-3, -1] | 0·301 |
|  | Control | -1 [-2, 0] | 0 [-2, 0] | 0 [-2, 1] |  |
| Propofol, mg/hr | Intervention | 150 [100, 240] | 100 [30, 200] | 180 [138, 255] | 0·825 |
|  | Control | 160 [105, 245] | 200 [170, 245] | 140 [120, 200] |  |
| Fentanyl, µg/hr | Intervention | 50 [50, 100] | 50 [38, 100] | 50 [50, 80] | 0·224 |
|  | Control | 50 [25, 100] | 25[25, 50] | 25 [25, 50] |  |
| Clonidine, µg/hr | Intervention | 60 [25, 95] | 60 [40, 85] | 50 [40, 120] | 0·996 |
|  | Control | 60 [40, 80] | 70 [55, 105] | 60 [50, 70] |  |
| pH | Intervention | 7·42 (0·07) | 7·40 (0·07) | 7·40 (0·07) | 0·922 |
|  | Control | 7·42 (0·08) | 7·40 (0·07) | 7·42 (0·06) |  |
| PaO_2_, mmHg | Intervention | 79 (13) | 82 (12) | 89 (14) | 0·255 |
|  | Control | 79 (15) | 79 (8) | 81 (19) |  |
| PaCO_2_, mmHg | Intervention | 45 (11) | 48 (9) | 48 (9) | 0·711 |
|  | Control | 44 (8) | 47 (7) | 46 (8) |  |

**Table E3:** **Sedation and gas exchange.** Data are presented as median [interquartile range] or mean (standard deviation). pH, PaO_2_ and PaCO_2_ are the closest values to the time point in each subject (at most 2 hours earlier or later) as we did not standardize arterial blood gas analysis in the protocol. In total, 15 patients received propofol (5 in the control group, 10 in the intervention group); 25 patients received fentanyl (12 in the control group, 13 in the intervention group); and 12 patients received clonidine (7 in the control group, 5 in the intervention group). * p-values for the two-way interaction term of group and time in the mixed linear models for the respective parameter. RASS = Richmond Agitation and Sedation Scale

|  | **Markers** | **Group** | **Time = 0hrs** | **Time = 12hrs** | **Time = 24hrs** | **p*** |
| --- | --- | --- | --- | --- | --- | --- |
| Endothelial function | vWF, ng/ml | Intervention | 3·83 [2·87, 5·10] | 4·16 [3·09, 5·31] | 4·11 [2·75, 5·19] | 0·785 |
|  |  | Control | 4·72 [3·24, 5·29] | 4·38 [3·38, 5·25] | 4·11 [2·82, 5·02] |  |
|  | ICAM-1, ng/ml | Intervention | 452 [271, 567] | 423 [269, 551] | 441 [285, 567] | 0·426 |
|  |  | Control | 466 [278, 949] | 419 [265, 1048] | 388 [245, 1168] |  |
|  | Ang-1, ng/ml | Intervention | 4·05 [2·08, 6·66] | 3·79 [3·03, 7·04] | 4·22 [2·01, 6·06] | 0·327 |
|  |  | Control | 4·14 [2·21, 7·38] | 3·71 [1·90, 9·20] | 4·73 [3·51, 6·90] |  |
|  | Ang-2, ng/ml | Intervention | 4·28 [2·99, 7·47] | 4·27 [3·17, 7·21] | 3·87 [2·96, 7·52] | 0·834 |
|  |  | Control | 4·66 [3·47, 8·03] | 4·96 [3·79, 6·34] | 4·09 [3·32, 5·50] |  |
|  | E-selectin, ng/ml | Intervention | 27·1 [16·4, 35·4] | 22·6 [17·8, 48·2] | 22·9 [16·8, 49·9] | 0·719 |
|  |  | Control | 32·1 [25·4, 48·5] | 33·0 [25·3, 46·7] | 35·2 [24·5, 47·5] |  |
|  | VCAM-1, µg/ml | Intervention | 2·06 [2·05, 3·70] | 2·72 [2·06, 4·36] | 2·71 [2·06, 5·36] | 0·719 |
|  |  | Control | 2·91 [2·16, 6·44] | 3·80 [2·09, 6·51] | 2·09 [2·06, 3·31] |  |
| Epithelial injury | RAGE, ng/ml | Intervention | 1·90 [1·45, 5·11] | 2·09 [1·44, 5·56] | 2·41 [1·08, 4·28] | 0·156 |
|  |  | Control | 1·54 [0·87, 2·94] | 1·63 [0·78, 2·86] | 1·23 [0·73, 2·65] |  |
|  | SP-D, ng/ml | Intervention | 30·8 [13·6, 58·7] | 23·1[9·5, 56·3] | 24·8[9·5, 52·6] | 0·369 |
|  |  | Control | 21·0 [13·4, 38·7] | 26·7 [12·1, 44·5] | 19·2[11·6, 36·8] |  |
| Inflammation | IL-6, pg/ml | Intervention | 39 [10, 67] | 22[8, 46] | 28 [9, 58] | 0·795 |
|  |  | Control | 27 [18, 42] | 28 [14, 49] | 21 [10, 27] |  |
|  | IL-8, pg/ml | Intervention | 19 [11, 36] | 25 [12, 39] | 26 [13, 50] | 0·423 |
|  |  | Control | 14 [9, 20] | 17 [10, 21] | 19 [11, 24] |  |
|  | IL-10, pg/ml | Intervention | 6 [3, 8] | 4 [3, 8] | 5 [3, 7] | 0·165 |
|  |  | Control | 4 [2, 6] | 3 [2, 4] | 3 [2, 5] |  |
|  | TNF-α, pg/ml | Intervention | 5 [3, 7] | 4 [3, 7] | 5 [3, 8] | 0·768 |
|  |  | Control | 6 [5, 7] | 6 [5, 7] | 6 [6, 7] |  |

**Table E4. Plasma protein biomarkers.** Data are presented as medians and interquartile ranges. Abbreviations: Ang-1, Angiopoeietin-1; Ang-2, Angiopoeietin-2; ICAM-1, soluble intercellular adhesion molecule-1; IL, interleukin; RAGE, receptor for advanced glycation end products; SP-D, surfactant protein D; TNF-α, tumor necrosis factor α; VCAM-1, vascular cell adhesion molecule 1; vWF, von Willebrand factor.*p-value for the two-way interaction term of group and time. Longitudinal differences between groups were not significant.

|  | **Overall (n=39)** | **Control (n=20)** | **Intervention (n=19)** | **p** |
| --- | --- | --- | --- | --- |
| Total duration of ventilation, days | 19 [11, 33] | 19 [11, 30] | 17 [11, 33] | 0·978 |
| Ventilation after the study, days | 5 [3, 16] | 4 [3, 18] | 7 [3, 14] | 0·673 |
| Ventilator-free days at day 28, days | 0 [0, 13] | 0 [0, 8] | 0 [0, 15] | 0·592 |
| Reintubation, n (%) | 13 (33%) | 7 (35%) | 6 (32%) | 1·000 |
| ICU mortality, n (%) | 7 (18%) | 6 (30%) | 1 (5%) | 0·111 |
| Hospital mortality, n (%) | 14 (36%) | 9 (45%) | 5 (26%) | 0·378 |

**Table E5: Patient-centered outcomes.** ICU: Intensive Care Unit.


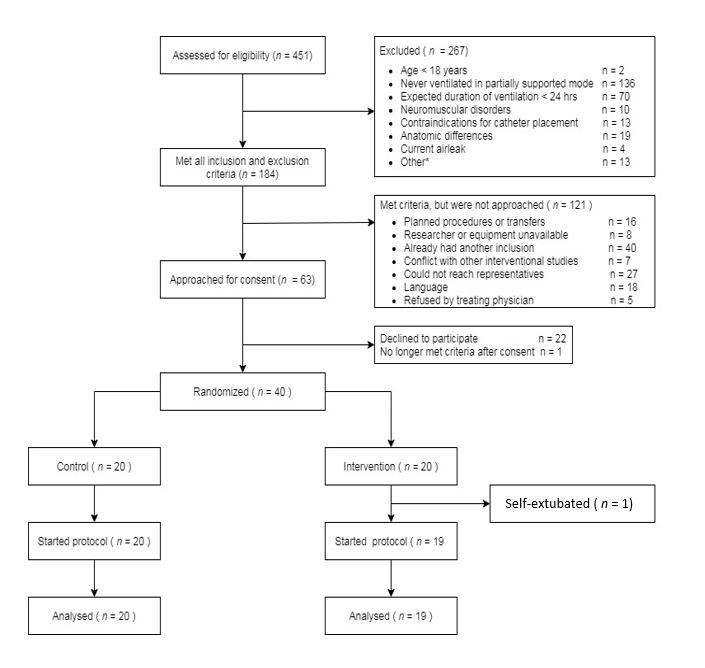


**Figure E1: Flowchart of patient screening and inclusion.** Unnamed other reasons for exclusion included: Infections with multi-resistant bacteria (n = 4), treatment restrictions (n = 6), patient on extra-corporeal membrane oxygenation (n = 1), screening failure (n = 2).

**A**

**B**


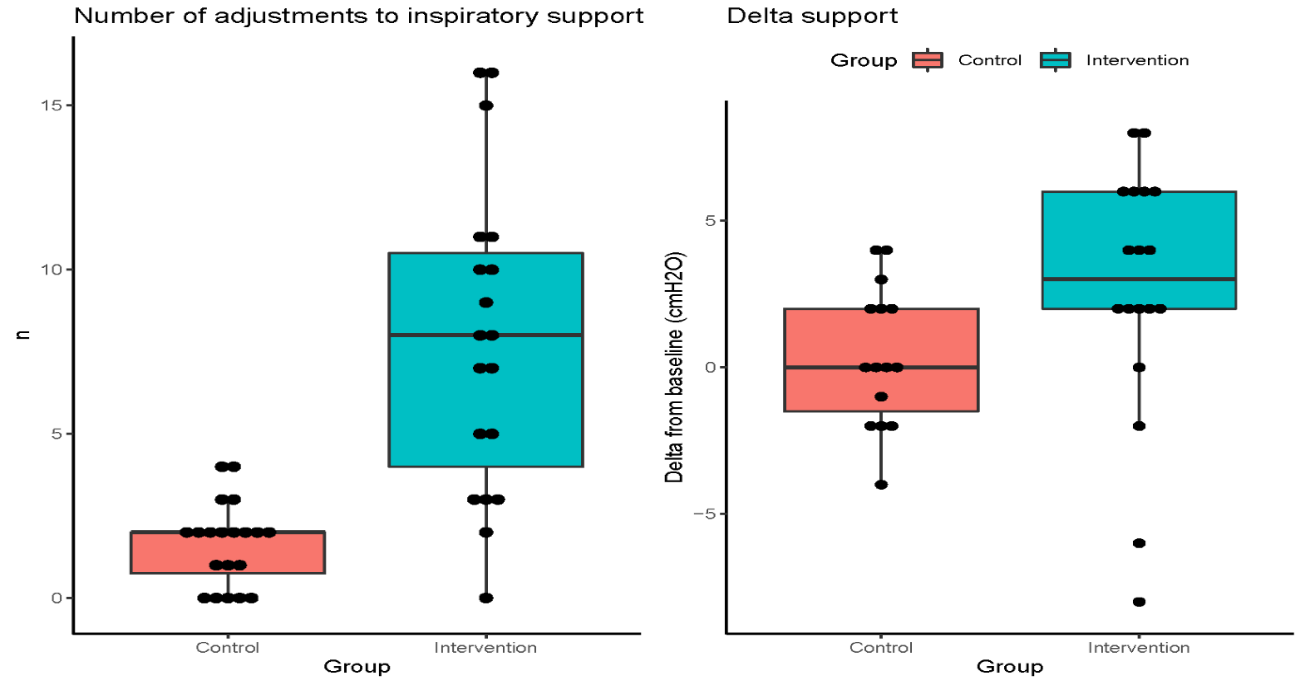


**C**


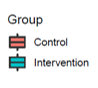
**
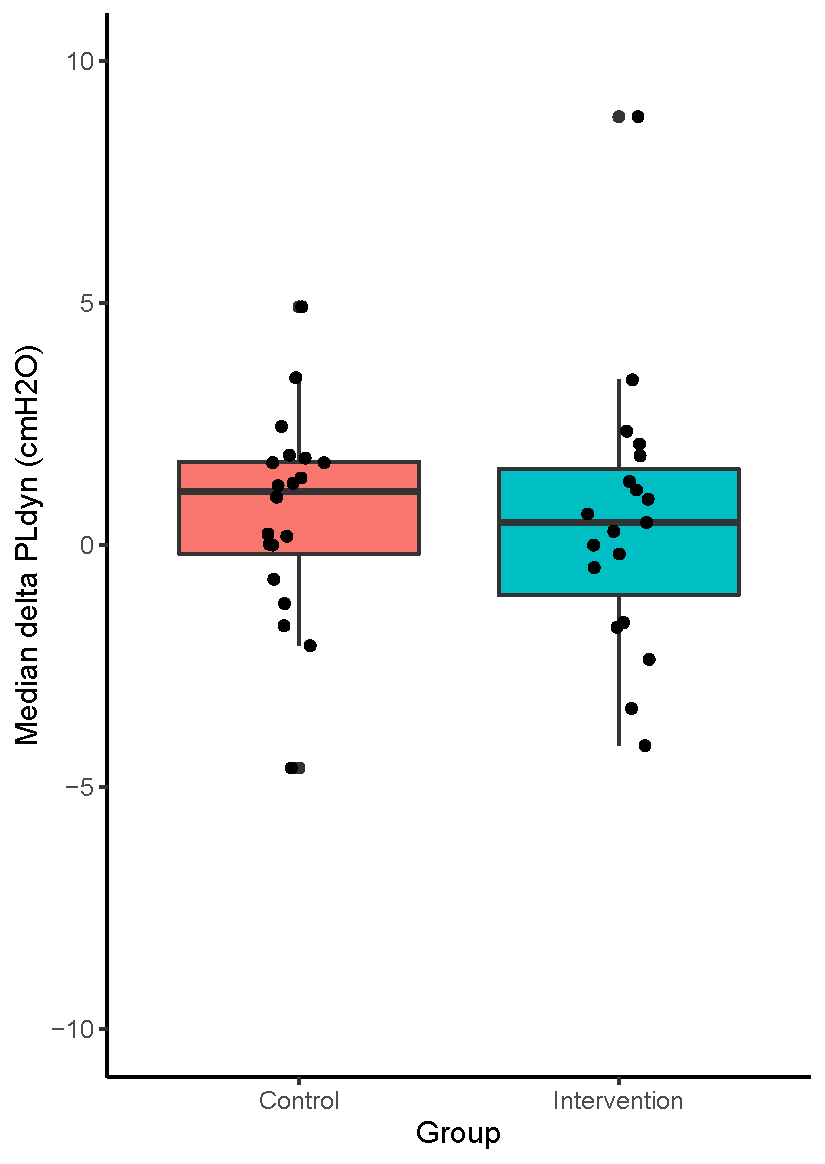
**

**Figure E2: Physiological analysis of support adjustments**. A: boxplot of the number of support adjustments per subject. B: Boxplot of the total difference in inspiratory support per subject. C: Boxplot of median change in dynamic transpulmonary pressure (PLdyn) per subject as compared to each subject’s baseline.


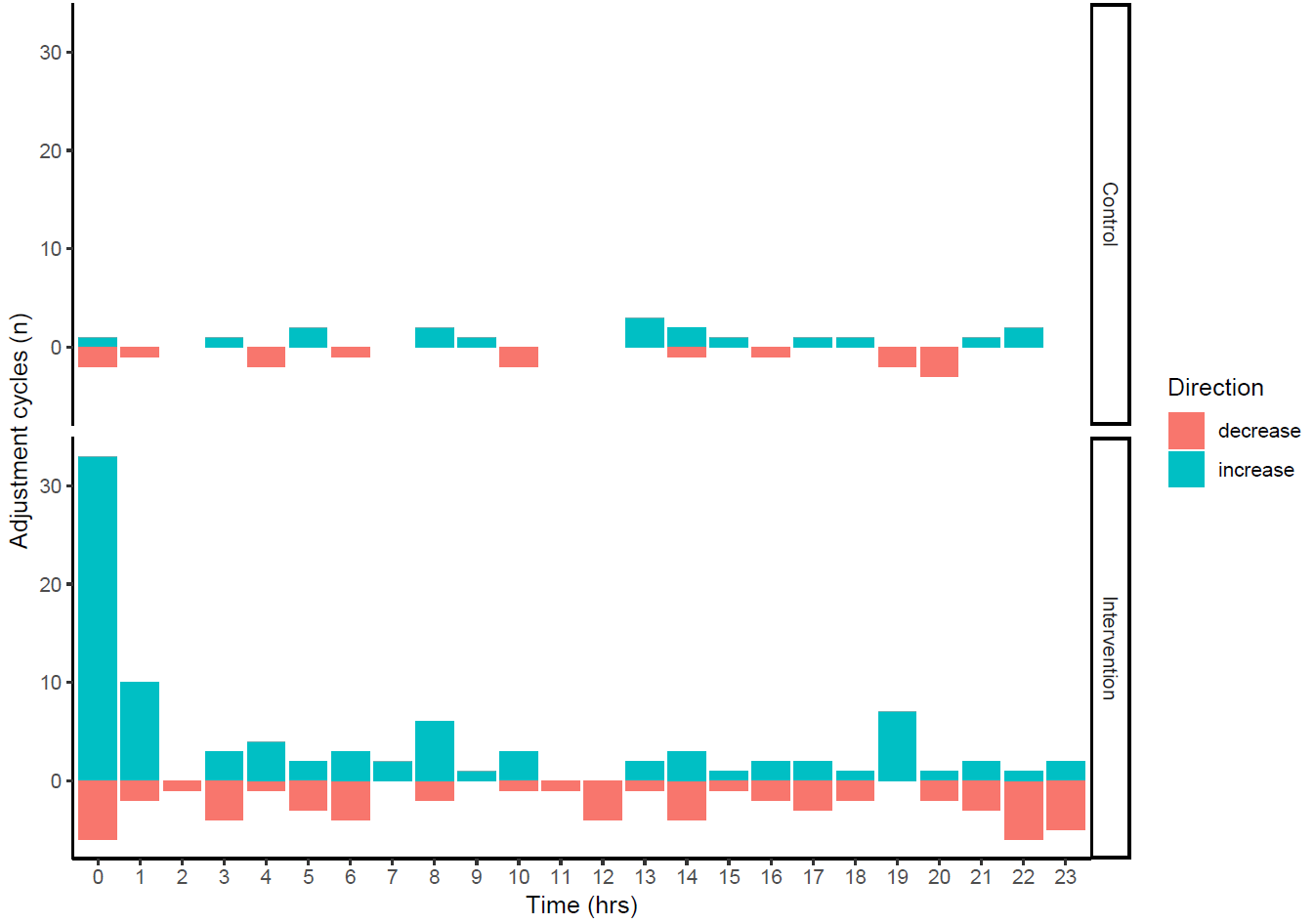


**Figure E3:** Amount and directions of support adjustments per hour in each group. ). In total, support was increased 91 times in the intervention group versus 18 times in the control group (p < 0.001). Support was reduced 58 times in the intervention group versus 15 times in the control group (p < 0.001).


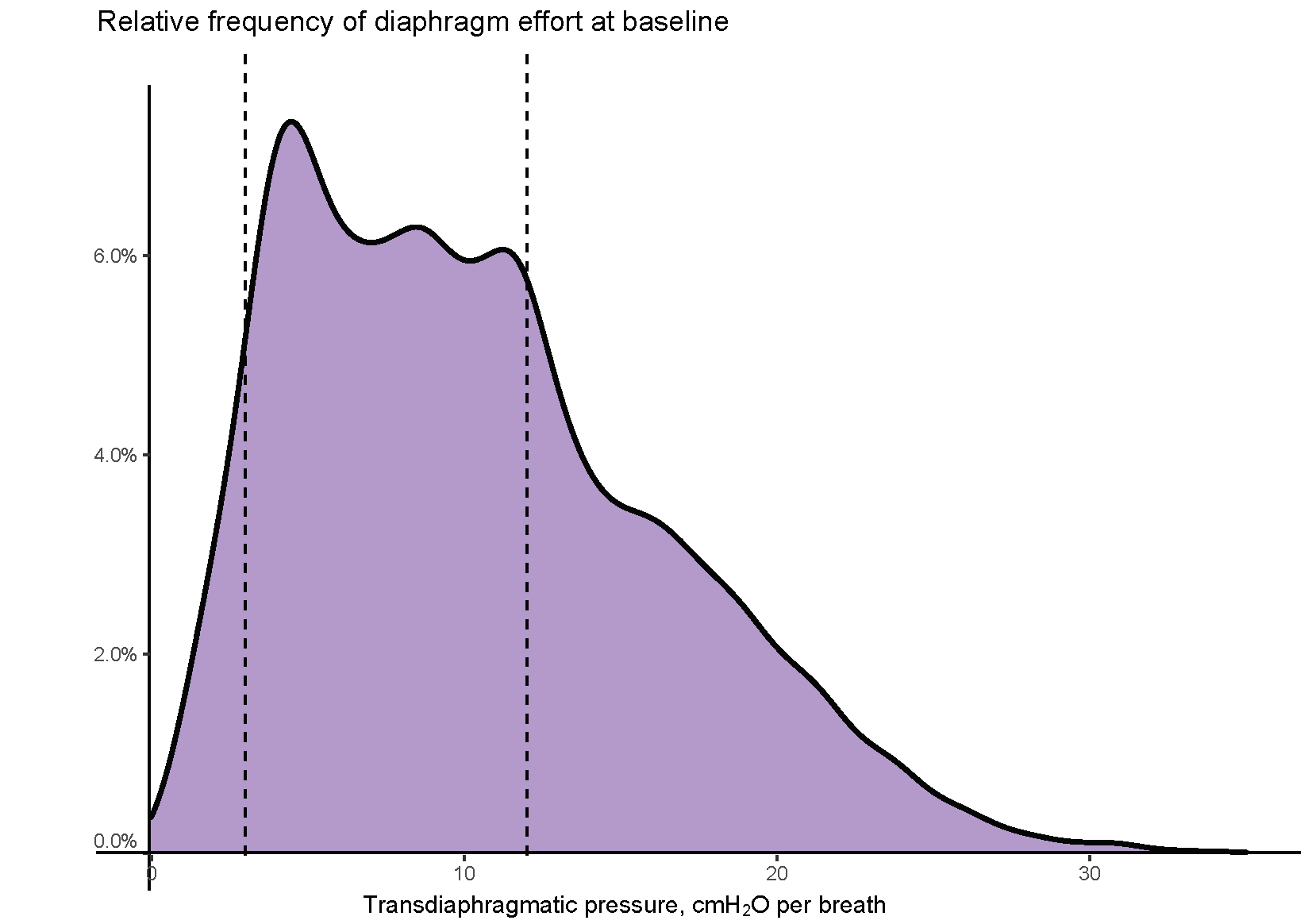


**B**

**A**


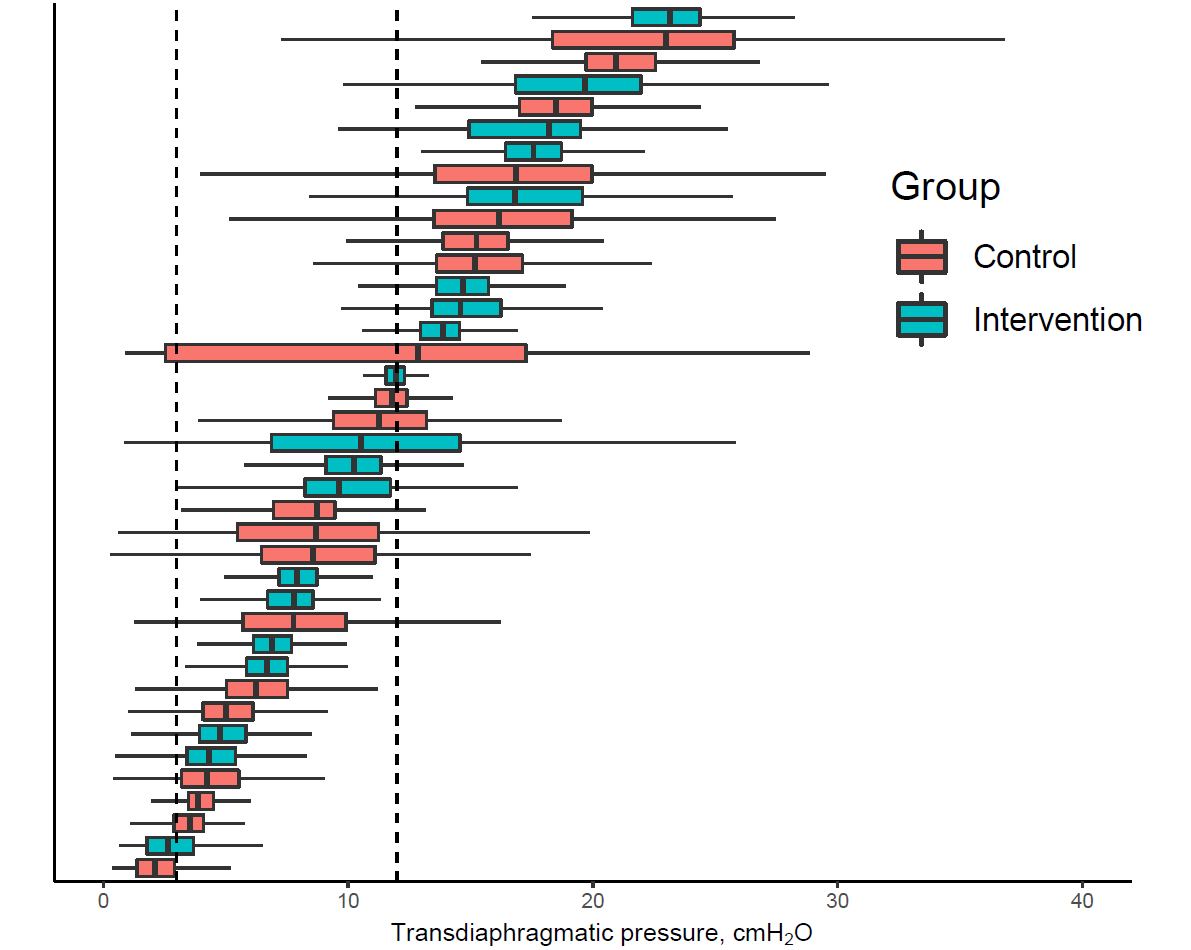


**Figure E4:** **Distribution of transdiaphragmatic pressure at baseline.** A) Density plot of diaphragm effort for the total study population. B) Boxplots of diaphragm effort per subject. The dashed vertical lines cross the x-axis at 3 cmH_2_O and 12 cmH_2_O, the predefined range of diaphragm-protective effort.


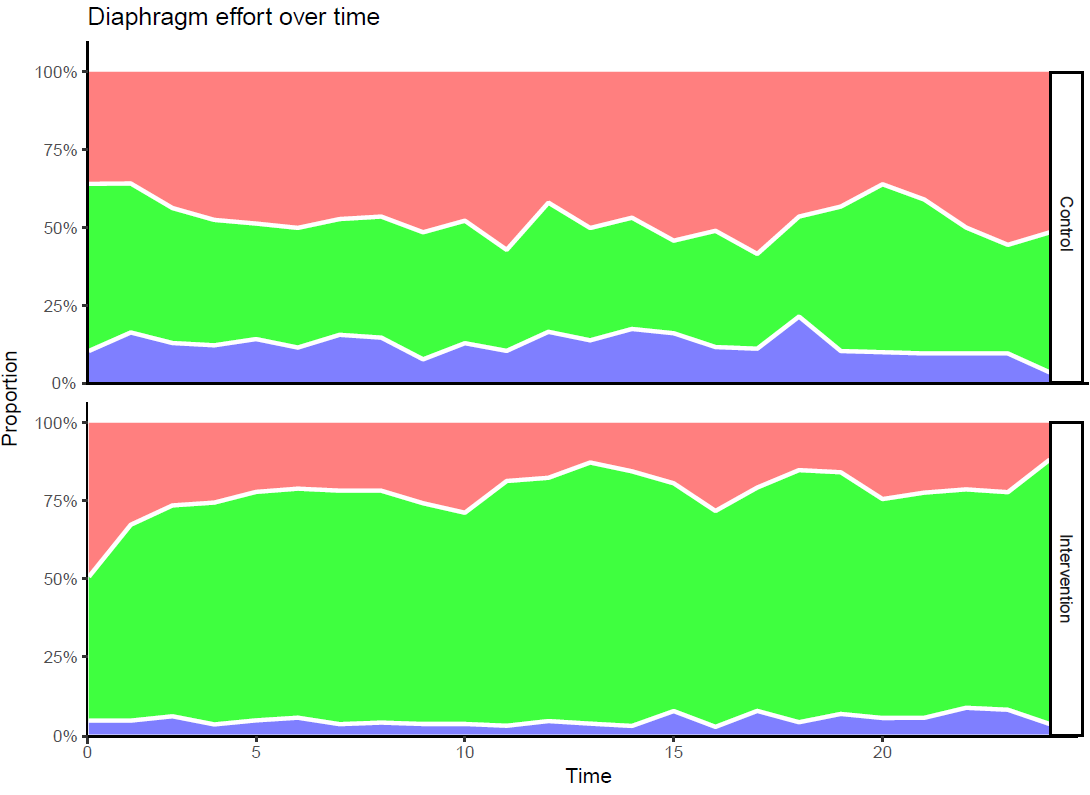


**Figure E5. Diaphragm effort over time.** Proportion of breaths with a transdiaphragmatic pressure below 3 cmH_2_O (blue), between 3-12 cmH_2_O (green), and above 12 cmH_2_O(red) over time per group.


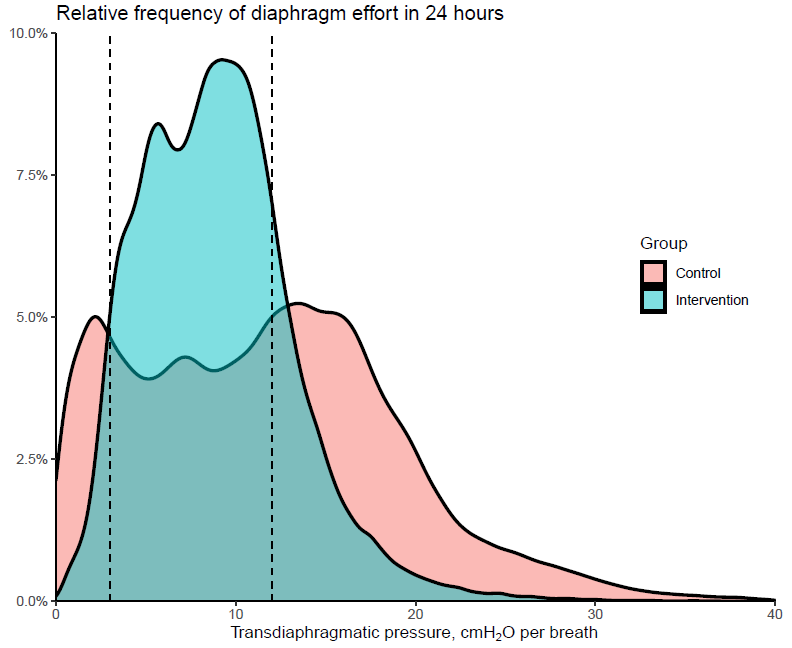


**B**

**A**


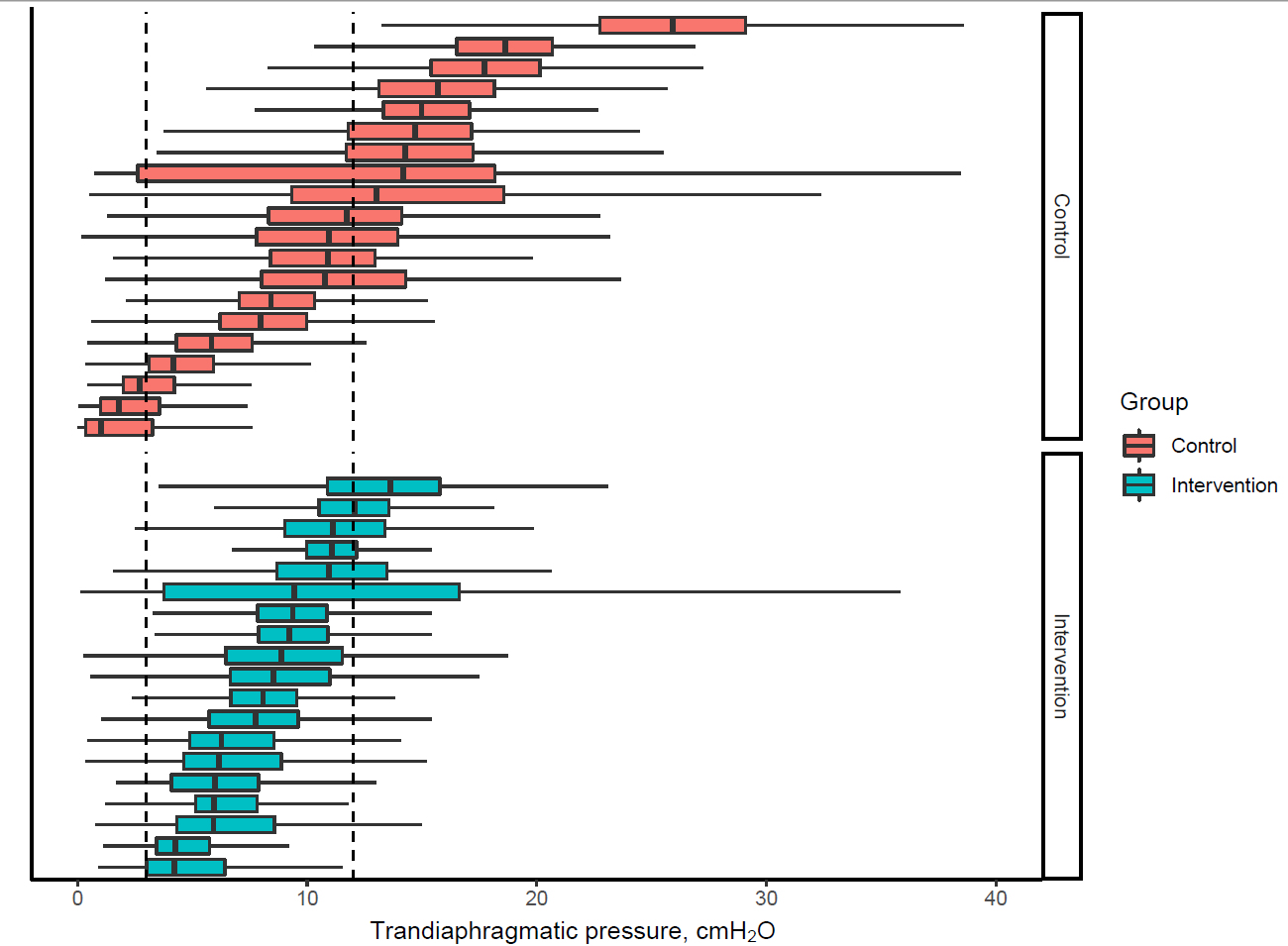


**Figure E6:** **Distribution of transdiaphragmatic pressure in the full study period.** A) Density plot for the control and intervention group. B) Boxplots for each subject. The dashed vertical lines cross the x-axis at 3 cmH_2_O and 12 cmH_2_O, the predefined range of diaphragm-protective effort.


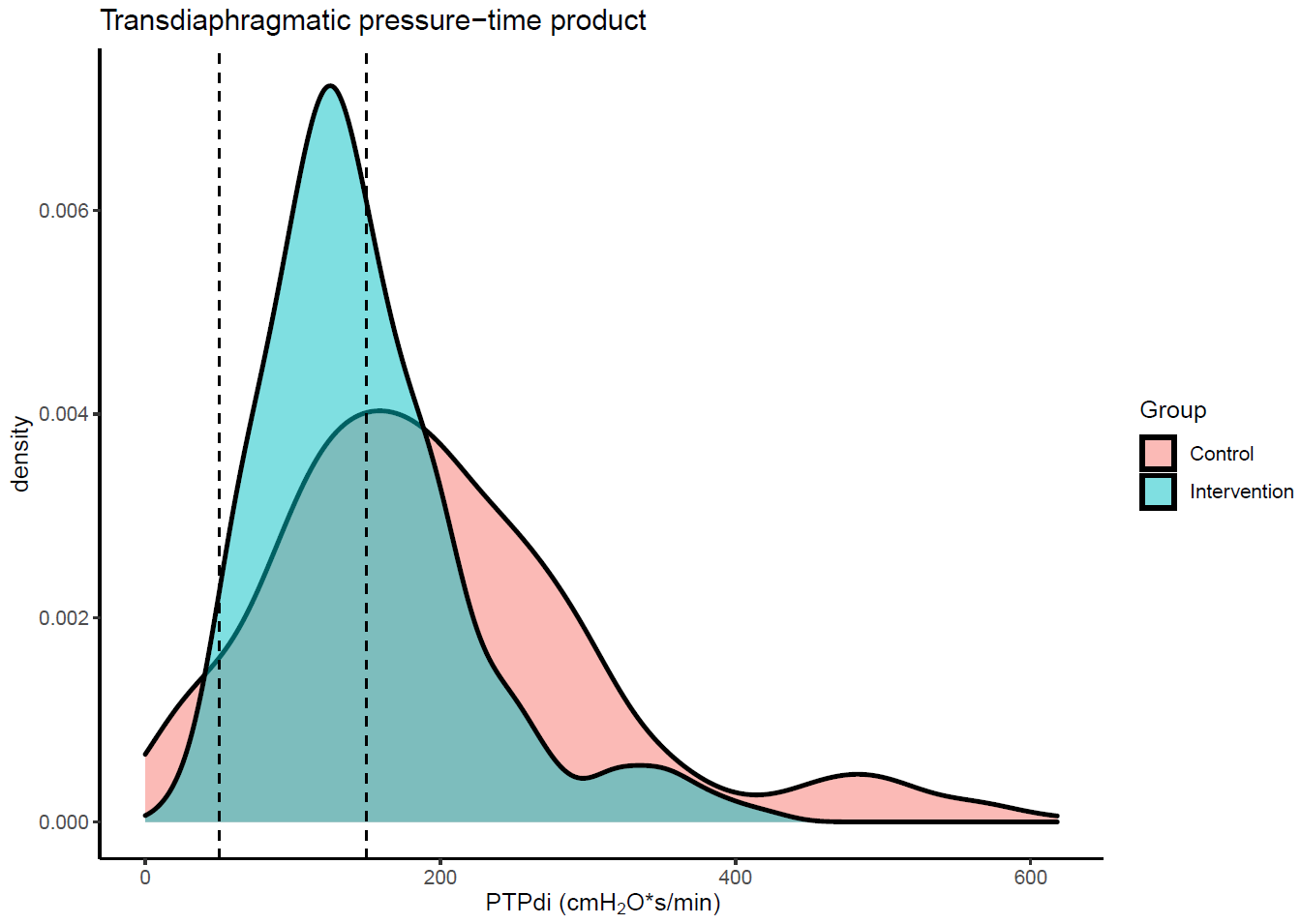


**Figure E7: Distribution of the pressure-time product of the diaphragm.** The dashed lines cross the x-axis at 50 cmH_2_O*s/min and 150 cmH_2_O*s/min, which is the range of diaphragm effort observed in healthy subjects during light exercise.(E6) The proportion of time that the pressure-time product of the diaphragm (PTPdi) was between 50 and 150 cmH_2_O*s/min was significantly higher in intervention group compared with the control group (57% versus 30%, respectively, difference in means 27%, 95% CI 20-33%, p<0.001).

**Supplementary results: breaths <8ml/kg PBW**

A recent statement has suggested that tidal volumes between 4-8 ml/kgPBW can be considered as lung-protective during partially supported mechanical ventilation.(E7) We analyzed the percentage of breaths below 8ml/kg and above 8ml/kg in each subject as a post-hoc analysis. Percentage was calculated as [breaths with Vt/PBW <8ml/kg] / [all breaths] * 100%.

**
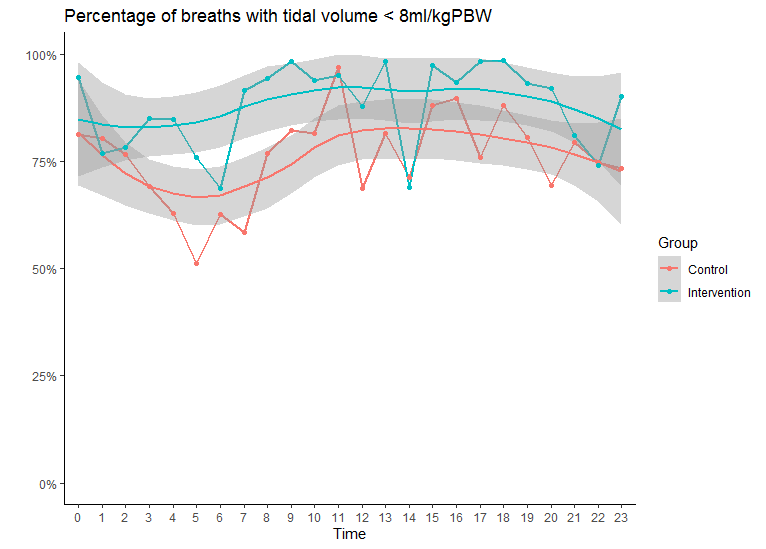
**

**Fig E8. Lung-protective breaths over time.** Proportions of breaths with tidal volume less than 8 ml per kilogram predicted bodyweight (ml/kg PBW) over time**.** Points show the observed median, the smooth line and shaded area represent the fitted mean and the 95% confidence obtained with local polynomial regression (Loess).

**Supplementary results: minute volume over time**


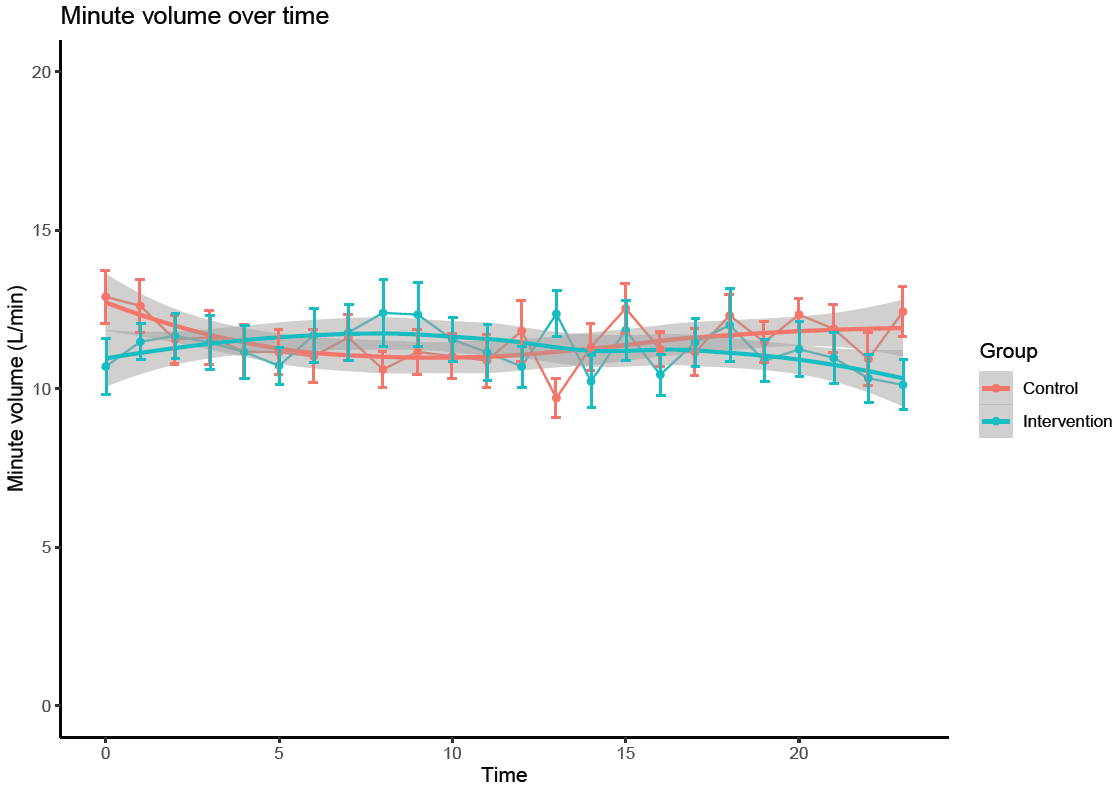


**Fig E9. Minute volume over time.** Dots represent the mean; bars represent the standard error of the mean; Shaded area represents the 95% confidence interval obtained with Loess-regression. None of the hours differed significantly between both groups in the post-hoc analysis (p = 0.146 for the interaction term of group and time).


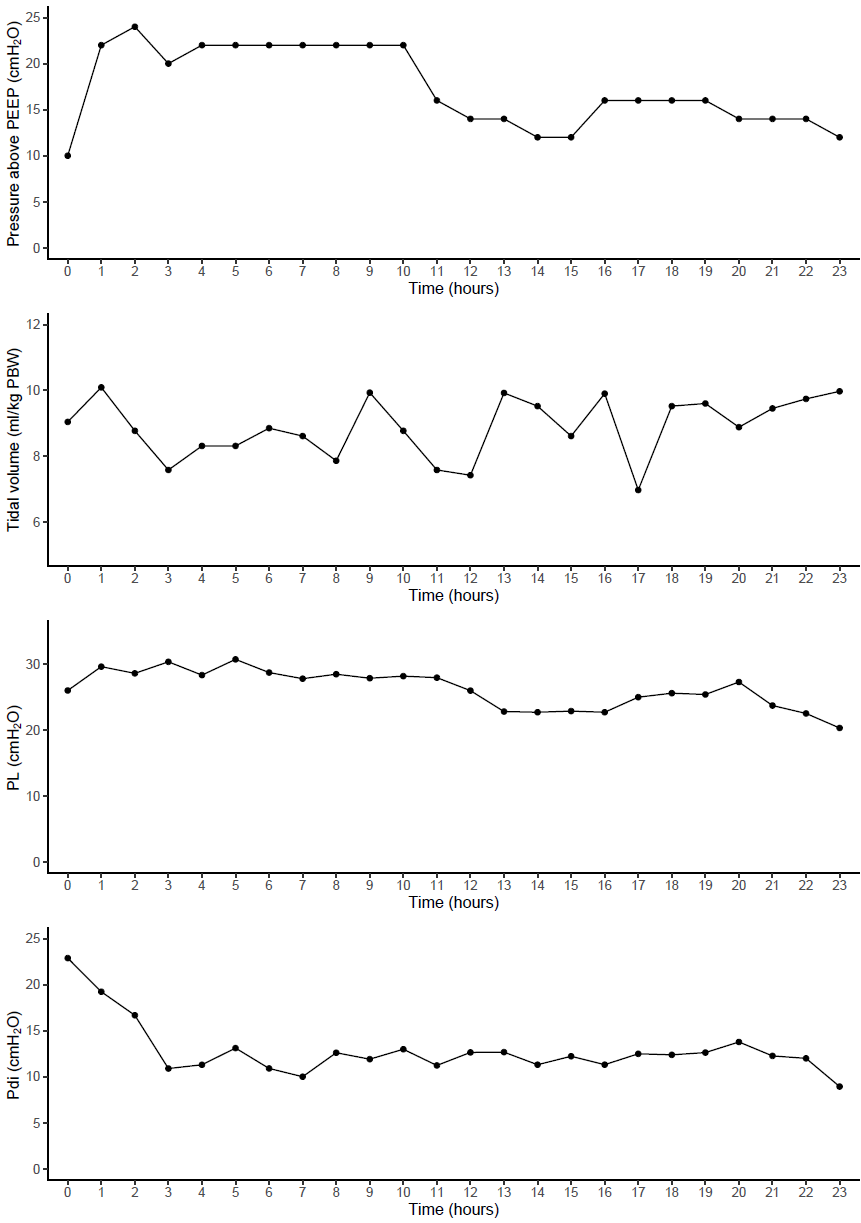


**Figure E10:** Inspiratory support level (Pressure above PEEP), tidal volume, dynamic transpulmonary pressure (PL) and transdiaphragmatic pressure (Pdi) in the participant with an adverse event. Although support was increased from 10 to 22 cmH_2_O, tidal volumes and dynamic transpulmonary pressure increased less dramatically because the participants’ own effort decreased substantially. Nevertheless, support titration was halted after discovering the emphysema. Tidal volumes had ranged from 8-10 ml/kg PBW in the days before inclusion (not shown). PBW, predicted bodyweight.

# **E-References**

E1. ATS/ERS Statement on respiratory muscle testing. [Internet]. *Am J Respir Crit Care Med* 2002; 166:518–624[cited 2017 May 2] Available from: http://www.atsjournals.org/doi/abs/10.1164/rccm.166.4.518

E2. Baydur A, Behrakis PK, Zin WA, et al.: A Simple Method for Assessing the Validity of the Esophageal Balloon Technique1–2 [Internet]. *Am Rev Respir Dis* 2015; 126:5–8[cited 2017 Apr 24] Available from: http://www.atsjournals.org/doi/abs/10.1164/arrd.1982.126.5.788

E3. Hess DR: Respiratory mechanics in mechanically ventilated patients. *Respir Care* 2014; 59:1773–1794

E4. Mauri T, Yoshida T, Bellani G, et al.: Esophageal and transpulmonary pressure in the clinical setting: meaning, usefulness and perspectives [Internet]. *Intensive Care Med* 2016; 42:1360–1373[cited 2017 Apr 17] Available from: http://link.springer.com/10.1007/s00134-016-4400-x

E5. Bhargava M, Wendt CH: Biomarkers in acute lung injury [Internet]. *Transl Res* 2012; 159:205–217[cited 2020 Dec 9] Available from: /pmc/articles/PMC4537856/?report=abstract

E6. Vries H de, Jonkman A, Shi Z-H, et al.: Assessing breathing effort in mechanical ventilation: physiology and clinical implications [Internet]. *Ann Transl Med* 2018; 6[cited 2019 Apr 9] Available from: https://www.ncbi.nlm.nih.gov/pmc/articles/PMC6212364/

E7. Goligher EC, Dres M, Patel BK, et al.: Lung- and Diaphragm-Protective Ventilation [Internet]. *Am J Respir Crit Care Med* 2020; 202:950–961[cited 2020 Nov 27] Available from: https://www.atsjournals.org/doi/10.1164/rccm.202003-0655CP
